# Investigating the effects of artificial intelligence on the personalization of breast cancer management: a systematic study

**DOI:** 10.1186/s12885-024-12575-1

**Published:** 2024-07-18

**Authors:** Solmaz Sohrabei, Hamid Moghaddasi, Azamossadat Hosseini, Seyed Jafar Ehsanzadeh

**Affiliations:** 1 Department of Health Information Technology and Management, Medical Informatics, School of Allied Medical Sciences, Shahid Beheshti University of Medical Sciences, Tehran, Iran; 2 Department of Health Information Technology and Management, Health Information Management, School of Allied Medical Sciences, Shahid Beheshti University of Medical Sciences, Tehran, Iran; 3 Department of English Language, School of Health Management and Information Sciences, Iran University of Medical Sciences, Tehran, Iran

**Keywords:** Breast cancer, Artificial intelligence, Deep learning, Precision oncology, Personalized breast cancer treatment

## Abstract

**Background:**

Providing appropriate specialized treatment to the right patient at the right time is considered necessary in cancer management. Targeted therapy tailored to the genetic changes of each breast cancer patient is a desirable feature of precision oncology, which can not only reduce disease progression but also potentially increase patient survival. The use of artificial intelligence alongside precision oncology can help physicians by identifying and selecting more effective treatment factors for patients.

**Method:**

A systematic review was conducted using the PubMed, Embase, Scopus, and Web of Science databases in September 2023. We performed the search strategy with keywords, namely: Breast Cancer, Artificial intelligence, and precision Oncology along with their synonyms in the article titles. Descriptive, qualitative, review, and non-English studies were excluded. The quality assessment of the articles and evaluation of bias were determined based on the SJR journal and JBI indices, as well as the PRISMA2020 guideline.

**Results:**

Forty-six studies were selected that focused on personalized breast cancer management using artificial intelligence models. Seventeen studies using various deep learning methods achieved a satisfactory outcome in predicting treatment response and prognosis, contributing to personalized breast cancer management. Two studies utilizing neural networks and clustering provided acceptable indicators for predicting patient survival and categorizing breast tumors. One study employed transfer learning to predict treatment response. Twenty-six studies utilizing machine-learning methods demonstrated that these techniques can improve breast cancer classification, screening, diagnosis, and prognosis. The most frequent modeling techniques used were NB, SVM, RF, XGBoost, and Reinforcement Learning. The average area under the curve (AUC) for the models was 0.91. Moreover, the average values for accuracy, sensitivity, specificity, and precision were reported to be in the range of 90-96% for the models.

**Conclusion:**

Artificial intelligence has proven to be effective in assisting physicians and researchers in managing breast cancer treatment by uncovering hidden patterns in complex omics and genetic data. Intelligent processing of omics data through protein and gene pattern classification and the utilization of deep neural patterns has the potential to significantly transform the field of complex disease management.

## Introduction

Humans have different genomes, live in different environments, and their physical responses to disease-causing factors and treatments vary. Consequently, standardized therapeutic approaches yield different outcomes in different individuals. Personalized Medicine involves a collection of activities and approaches for appropriate disease management, considering individual-specific characteristics. It provides personalized treatments based on the genomic characteristics of individuals [1] Therefore, the foundation of personalized medicine lies in the identification and classification of individuals and therapeutic methods based on their genetic traits. Thus, personalized medicine can be defined as medicine based on genomic characteristics [2]. Breast cancer is a complex genetic disease caused by genetic mutations [3]. Mutation patterns can vary from one tumor region to another and change over time. This process leads to the creation of genetically distinct subpopulations of cancer cells, which can result in drug resistance in patients [4]. Therefore, identifying molecular differences between tumors is a crucial aspect of precise oncology for selecting the most effective treatment [5]. Precision oncology aims to personalize the therapeutic regimen for each patient based on accurate evaluation of cancer progression or the risk of recurrence, with the goal of achieving effective treatment. Accurately predicting which patients will respond to treatment before they undergo it is a key objective. For example, in breast cancer, the status of the hormone receptor ER is a good indicator of treatment response, but resistance, both intrinsic and acquired after therapy, is common. Therefore, selecting an effective life-saving treatment for the patient is crucial [6]. The study of human genes and proteins (multi-omics) and artificial intelligence are two potential technologies that can transform cancer treatment through precise oncology-guided personalized treatment selection to achieve effectiveness. For example, chemotherapy is the main treatment used for metastatic breast cancer, but the sensitivity and response of different patients to it vary [5–7]. For some individuals, it has a significant impact, while for others, its effect may be minimal or non-existent [8, 9]. The ability to predict who will respond to treatment allows for the use of treatment for those who will benefit the most from it. Patients who are not likely to respond can receive alternative treatments and avoid poisoning and side effects of unnecessary drugs [10, 11]. The emerging technologies that investigate the genome and cancer molecules enable scientists to study approximately 500 genes for selecting appropriate treatment in a cancer patient. However, their challenge lies in examining 500 genes in multiple patients, where all their genes change over time, making it complex [12]. This is where artificial intelligence can be effective in discovering patterns of genetic data behavior changes in patients and predicting drug resistance and the protein and cellular mechanisms leading to this resistance. It can help prevent unnecessary drug toxicities and assist oncologists in using expensive drugs when necessary, which result in effective cancer treatment [13]. Moreover, it can prevent unnecessary invasive biopsy, which is a method of guiding cancer cells’ DNA invasion into the bloodstream, which has adverse effects [14, 15]. The main objective of this study is to review the applications of AI algorithms and their effectiveness in personalized medicine approaches. The main objective of this study is to reflect on various machine-learning methods in breast cancer detection and the effectiveness of artificial intelligence applications in precise oncology with the aim of personalized disease management. This investigation can assist scientists and physicians in selecting techniques that have proven to be highly accurate in personalized breast cancer management. They can also have a comprehensive perspective on the personal medical applications in the diagnosis, treatment, and screening of breast cancer.

## Materials and methods

The present study is a systematic review based on the PRISMA checklist 2020 [16]. We know that in evidence-based medical research, formulating research questions is considered the most important part of these studies.

### Eligibility criteria

Therefore, in this study, the SPICE tool [17], which is a step-by-step framework for formulating questions to find evidence in research, was used. SPICE expands on the PICO acronym (Population, Intervention, Comparison, and Outcomes) in two distinct manners. Firstly, the population component is divided into setting and perspective components. Secondly, the term “outcomes” is substituted with “evaluation” to foster a more comprehensive evaluation framework and merge concepts such as “outputs” and “impact” into one holistic perspective. Efforts were made to select studies from around the world that had used artificial intelligence in personalizing breast cancer management (Setting & Intervention). In these studies, breast cancer patients had benefited from personalized treatment (precision oncology). Ultimately, artificial intelligence had provided a favorable impact on personalizing breast cancer patient management (Evaluation). Considering specific scopes for further exploration, the following questions were designed:

1. What are the applications of artificial intelligence in precision oncology of breast neoplasms?
2. Which intelligent artificial intelligence techniques have been used in precision oncology of Breast cancer?
3. What are the reported effects of artificial intelligence methods, using which indicators, on? Personalizing breast cancer management?

### Including and excluding criteria

In order to have a more accurate response to the research questions, certain criteria were considered for selecting articles to be studied. These criteria included: (I) only the article were used, (II) focusing on the investigation, prediction, treatment, screening, and early detection of breast cancer, (III) studies that were based on omics datasets.

Additionally, certain criteria were considered for excluding articles from the study, such as: (I) articles that were not relevant to personalized management of breast cancer, (II) studies that were not in the form of articles (books, conference abstracts), (III) studies where the modeling methodology was not fully explained.

### Information sources and search strategy

After determining the research questions, a systematic search was conducted in databases such as PubMed, Web of Science, Scopus, and Embase, for relevant articles published between the years 2015 and 2023, using keywords present in the title, abstract, mesh terms, and key terms. The final search was conducted on January 31st, 2023. The search strategy, and the mesh and emtree terms are presented in the Table 1. The search was performed by combining these two groups of words and using the boolean AND operator. Shortening techniques, phrase search and other related techniques were used in order to conduct a comprehensive search.

**Table 1:** Vocabulary search formula in databases

Search Mesh term and formula
I: (Breast Cancers OR Breast Malignancy OR Malignancies OR Neoplasms OR Breast Benign Neoplasms OR Breast Benign Neoplasm OR Tumor)
II: (Diagnosis OR Prognosis OR Predictive OR Screening OR treatment)
III: (Machine Learning OR Deep Learning OR Artificial Intelligence OR machine intelligence OR Knowledge acquisition)
IV: (Precision oncology OR Personalized oncology OR Personalized cancer treatment OR Precision Medicine)
Search strategy: I AND II AND III AND IV
**PUBMED**: (“Neoplasm” OR “Tumors” OR “Tumor” OR “Breast Cancer” OR “Breast Cancers” OR “Malignancy” OR “Malignancies” OR “Malignant Neoplasms” OR “Malignant Neoplasm” OR “Neoplasm, Malignant” OR “Neoplasms, Malignant” OR “Benign Neoplasms” OR “Neoplasms, Benign” OR “Benign Neoplasm” OR “Neoplasm Benign”) AND (“Artificial Intelligence” OR “Computational intelligence” OR “machine intelligence” OR “Knowledge acquisition “) AND (“Precision Medicine” OR “Personalized medicine” OR " Personalized oncology “)

### Screening phase

In the screening phase, both authors (S.S and S.J.E) reviewed the articles based on their titles, abstracts, and eliminated irrelevant articles. In the next phase, the full text of the selected articles was evaluated separately by the two authors using entry/exit criteria. In cases where there was disagreement between the two authors, the issue was resolved through intellectual brainstorming and consensus with the help of a third author (H.M). In the data extraction stage, artificial intelligence models were used to analyze the precise oncology data of breast cancer and performance indices of the models were extracted. The screening methods were performed based on the PRISMA 2020 approach. The quantitative analysis of the data was conducted in the statistical software R. The first author’s name, year, and place of publication of the article were also extracted. Finally, the obtained results were presented in Table 2.

### Study risk of bias assessment

To address bias, the Critical Appraisal Checklist from the Joanna Briggs Institute (JBI) [18] was used to evaluate the risk of bias in cross-sectional analytical studies. The checklist was completed by two authors, and in case of disagreement between the two authors, the disagreement was resolved through discussion with the third author. The aim of this evaluation is to appraise the methodological excellence of investigations and comprises seven inquiries in the following order: (1) Were the standards for inclusion in the sample explicitly defined? (2) Were the subjects of the study and the setting comprehensively portrayed? (3) Was the exposure gauged in a legitimate and dependable manner? (4) Were objective, established standards utilized for the measurement of the condition? (5) Were confounding factors recognized? (6) Were approaches to handle confounding factors specified? (7) Were the outcomes gauged in in a valid and reliable way. These inquiries can be addressed employing four alternatives: (1) yes; (2) no; (3) unclear; and (4) not applicable. Each yes response corresponds to one score, and if 70% of the inquiries are responded to “yes” in a study, the risk of partiality was judged to be “low.” If 40 -69% of the inquiries were answered “yes”, the risk of partiality was deemed “moderate,” and below 40% was considered “high risk.”

### Processes used to decide which studies were eligible for each synthesis

In this systematic review, the results of studies in which the performance of artificial intelligence techniques were reported quantitatively with indicators of precision, accuracy, specificity, sensitivity, AUC (area under the ROC curve) [19], in order to measure the effect of using Artificial intelligence in the personalized management of breast cancer was investigated.

## Results

**Fig. 1:**
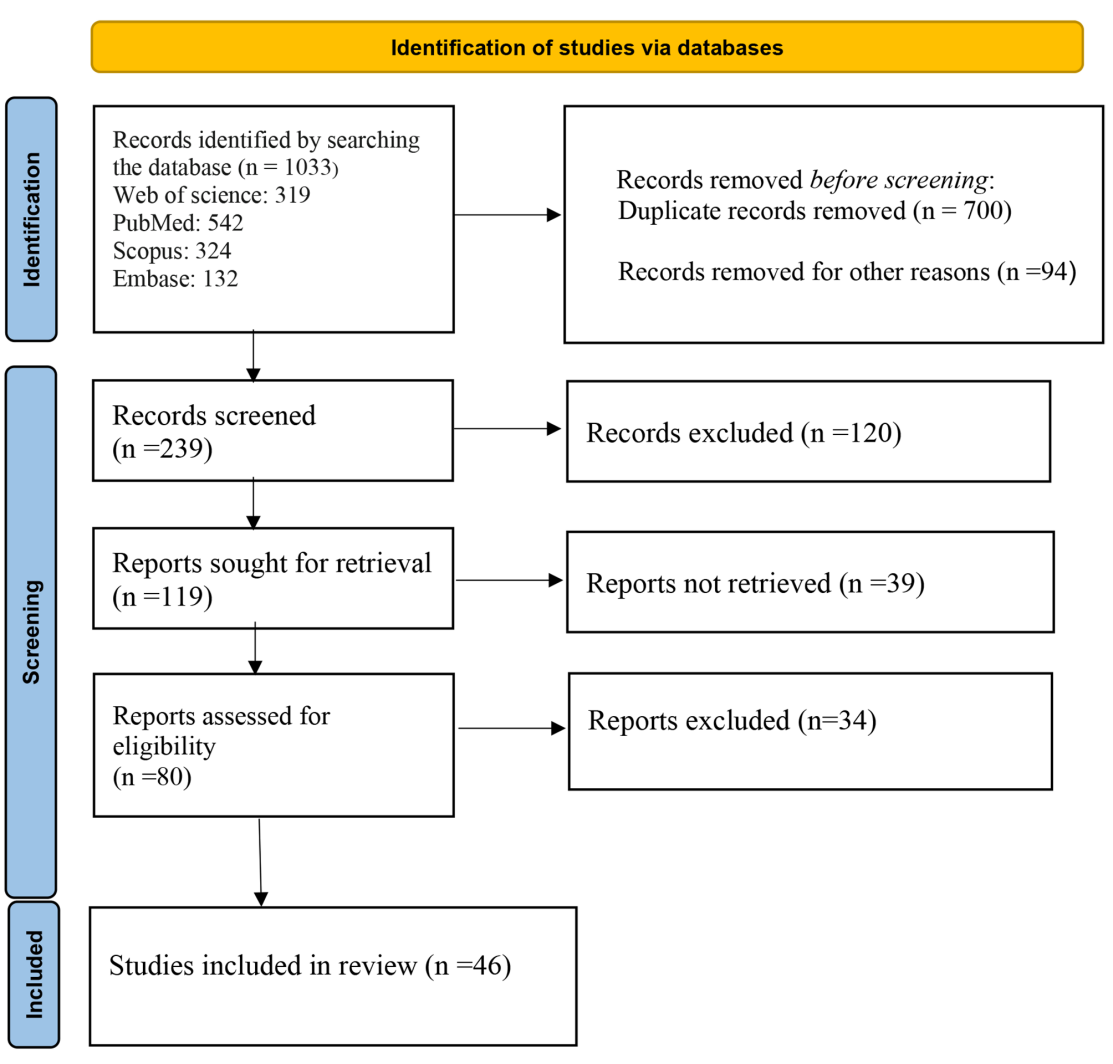
PRISMA flow diagram

As shown in Fig. 1, the database search resulted in the retrieval of 1,033 records until September 2023. After removing duplicate studies and reviewing based on entry indices to the study, ultimately 46 articles that met the entry conditions were selected for review, the specifications of which are mentioned in Table 2. The conducted studies indicate that the data used for modeling through machine learning has had a high diversity. For example, 59% (27 articles) of the reviewed articles used patient medical record data as input, and in four articles (1.847%), biological samples such as genes, molecular samples, and cell classes were reported. In 14 articles (30.4%), genomic data such as gene expression, genetic mutation data, phenotype data, proteomics were used with drug response data as input in artificial intelligence methods. In 12 articles (5.52%), radiomic data (radiography with biological indicators) and in three articles (1.38%), radiogenomic data were used by researchers for the management of neoplasm treatments. However, in 24 articles (52.3%), drug response data was used, indicating the necessity of considering different data dimensions in creating personalized management of breast cancer. The effectiveness of the selected artificial intelligence methods in different studies was examined and is shown in Table 2. The performance of the used methods was evaluated and selected with various indices, including accuracy, precision, sensitivity, feature, AUC. The reported indices showed that the performance of the used methods is at a significant level. Therefore, many of the algorithms used in the studies indicate the ability of artificial intelligence in early detection, predicting response to treatment, patient survival, and screening. Ultimately, the reviews showed that six studies using various artificial intelligence algorithms such as SVM, DNN, ANN, CNN on multi-omics data, one study using ANN, DNN models on omics data, also 10 studies using CNN, DT, XGB, MLP methods on genomics data, 14 studies mostly using SVM, XGB, CNN, RF methods on radiomics, five studies with high frequency using CNN methods on radiogenomics data, 6 studies mostly using RF, CNN algorithms on pharmacogenomics data, two studies using SVM and RF on proteomics data, one study using linear MSKCC model on epigenetic data and one study using GB, XGB, RF on transcriptomic data have achieved acceptable results (indices above 80%).

**Table 2:** Characteristics of the reviewed articles in the present systematic study

Names of authors Country, year of publication	Type of disease management	Artificial intelligence methods	Software	Input	Data set	Effectiveness	Outcome
Tong L et al. [20] USA 2020	Prediction of patient survival	Convolutional neural network	Python	Multi-omics	TCCA TCG	AUC= 0.72 sen=90% Spe=88%	Successful prediction of survival of patients with a high percentage of indicators
Lee M.K et al [21] Korea 2020	Prediction of patient survival	SVM NB RF	R	Proteomics	Local genomic and clinical data	AUC =0.912 AUC =0.791 AUC =0.978	Accurate classification of breast cancer type
Amiri Souri E et al. [22] England 2021	Prediction of patient survival and classification of tumor type	XGBoost KNN DT	Python	Genomics	GPL570, GPL96 (5031 breast cancer + 12,608 genes)	AUC=0.84 Acc=89% Spe=83%	Successfully predicting the survival of patients with a high percentage and achieving a successful classification in determining the type of breast cancer category
Sharma S et al. [23] Netherland 2020	Prediction of patient survival	VGG16, VGG19, ResNet50,SVM	Python	Genomics	Pathology of breast cancer patients	AUC =0.95 AUC =0.93 AUC =0.94 AUC =0.95 Acc= 93.97%	Achieving the highest level of indicators in breast cancer diagnosis
Sammut SJ et al [24] England 2021	Predicting response to treatment	SVM LR	Python	Multi-omics	DNA+RNA clinical pathology data	AUC =087 AUC =0.70	High accuracy to detect the response rate to treatment
Meti N et al. [25] USA 2021	Predicting response to treatment	MLR, RF, k-NN, NB, SVM	IBM Spss	Genomics	Hospital data of breast cancer patients	Sen=70.7% Spe=84% AUC =0.88	The classifiers showed the best prediction performance among all models.
Nguyen LC et al. [26] France 2021	Predicting drug response	RF, RF-OMC	Python	Pharmacogenomics	The NIBR-PDXE+ 26 database	AUC =0.84 AUC =0.65 SPE=100%	High accuracy to detect the level of response to the drug
Ramkumar, C. et al. [27] USA 2018	Prognosis of breast cancer	RF, SVM, Elastic Net (ESL), RBF, MLP, RBF-SVM	-----	Proteomics	Data of 268 breast cancer patients	AUC=0.67 SPE=90%	Achieving the highest level of indicators in the prognosis of breast cancer
Brocato TA et al. [28] USA 2017	Response to neoadjuvant treatment of breast cancer	regression line, HALO AI classifiers	MATLAB	Genomics	3990 histological pathology images	AUC=0.80	Significant efficacy of patients achieving pathologic complete response (PCR).
Roy S et al. [29] USA 2017	A clinical radiomic signature of FDG PET in predicting response to neoadjuvant chemotherapy	CART SVM NB RBA	MATLAB	Radiomics	TNBC patients, CCDB	ACC=80%, ,75%, 78%,74%	High efficiency in identifying radiomic signatures of FDG-PET (RadSig) for predicting and evaluating response to treatment
Mehmood A et al [30] China 2023	Predicting drug response	Elastic Net, LASSO, and Ridge regression	R	Pharmacogenomis	GDSC29 CCLE	AUC= 0.890	High efficacy in predicting treatment response
Farahmand S et al [31] USA 2023	Prediction of breast cancer	CNN	Python	Genomics	TCGA	AUC= 0.82	High performance in predicting breast cancer
Webber JT et al [32] USA 2018	Predicting drug response	Elastic net, RF, SVR	Python	Pharmacogenomics + Omics	TCGA, METABRIC	SEN= 90%	High efficiency in predicting the response to the drug
Li F et al. [33] China 2021	Predicting response to chemotherapy	CNN	Python	Genomics	Pathology images	AUC=0.853	High performance in predicting response to chemotherapy drug
Bitencourt, A.G et al. [34] China 2022	Predicting response to chemotherapy	RF SVM	MATLAB	Radiomics	Radiology and genetics images	ACC=85.1% SEN=87% SPE= 81%	Achieving high indices in predicting response to chemotherapy
Orozco JI et al [35] USA 2022	Classification of primary tumors of breast cancer	MSKCC model	Python	Epigenetics	TCGA	AUC =0.88	High performance in classifying primary breast cancer tumors
Gupta S et al [36] USA 2017	Survival prediction of breast cancer patients	CNN ANN RBM Deep Autoencoders	Python	Omics	Hospital data of breast patients	ACU=97%, 91%,96%, 89%	Achieving high indicators in predicting patient survival
Malik V et al [37] USA 2017	Prediction of survival and response to drugs in breast cancer patients	ANN K-means	MATLAB	Multi-omics	TCGA GDSC	ACC= 94%	Achieving high indicators in predicting patient survival
Hoang DT et al. [38] USA 2022	Prediction of response to treatment of breast cancer patients	ResNet50, ENLIGHT CNN, DeepPT	Python, R	Genomics	TCGA	SEN=68%	Prediction of response to treatment of breast cancer patients with high accuracy
Mourragui SM et al [39] Netherland 2021	Prediction of response to drug therapy in breast cancer patients	deep learning regression (DL) KRR, ComBatþDL, ElasticNeT, PRECISE	Python	Genomics	TCGA HMF GDSC	AUC=0.99,0.97,0.91,0.93 ACC= 89% SEN=80%	High performance in predicting response to chemotherapy drug
Kuenzi BM et al [40] USA 2020	Predicting response to drug therapy in breast cancer patients	VNN CNN	PyTorch	Pharmacogenomic	CTRPM GDSC	AUC= 1 SEN=70% ACC=78%	Accuracy and high efficiency in predicting drug response
Sharifi-Noghabi H et al [41] Canada 2021	Predicting response to drug therapy in breast cancer patients	CAN, Transfer learning	Scikit-learn and Scipy Python	Omics	GDSC PDX TCGA	AUC=0.980	High performance in predicting response to chemotherapy drug
Sharifi-Noghabi H, et al [42] Canada 2019	Predicting response to drug therapy in breast cancer patients	DNN	R	Multi-omics	GDSC CCLE TCGA	AUC= 0.806	Achieving high indicators in predicting response to chemotherapy drugs
Liu Q et al. [43] USA 2019	Predicting response to drug therapy in breast cancer patients	SVM-RRF SVM BSBM SBC	----	Multi-omics	GSE17705 GDSC	AUC= 0.94	High performance in predicting response to chemotherapy drug
Sammut SJ [44] England 2022	Breast cancer diagnosis	CNN MobileNetV3 ResNet-101 ResMLP	Python	Multi-omics	Hospital data of breast cancer patients	AUC= 0.87	Acceptable efficacy in breast cancer diagnosis
Saha A et al [45] USA 2018	Prediction of breast tumor behavior with radiogenomics	machine learning-based multivariate models	MATLAB	Radiogenomics	Hospital data of breast cancer patients	AUC=0.70	High performance in predicting tumor behavior
McAnena P et al [46] Ireland 2022	Classification of response to chemotherapy of breast cancer patients	LASSO SVM	R	Radiomics	Hospital data of breast cancer patients	AUC= 0.81	High performance in predicting response to chemotherapy drug
Li Q, et al [47] China 2022	Prediction of response to neoadjuvant chemotherapy	SVM KNN DT	Python	Radiomics	Hospital data of breast cancer patients	AUC= 0.84 ACC=75%	Accuracy and high efficiency in predicting response to chemotherapy drugs
Bitencourt AG et al. [48] USA 2020	Prediction of pathological condition of patients after neoadjuvant chemotherapy	DT	MATLAB	Radiomics	Hospital data of breast cancer patients	AUC= 0.761	High indices in predicting response to chemotherapy drugs
Yu Y et al [49] China 2021	Prediction of metastasis of tumors in breast cancer patients	SVM	Python	Radiomics	Hospital data of breast cancer patients	AUC=0.93 SEN=75% SPE=68%	Achieving high indicators in the successful prediction of disease metastasis
Vigil N et al [50] USA 2022	Breast cancer diagnosis	Deep Learning model-made Radiomics RF	Python	Radiomics	Hospital data of breast cancer patients	ACC=85% AUC=0.90	Accuracy and high efficiency in cancer diagnosis
Militello C et al [51] Italya 2022	Prediction of malignant masses in breast cancer	UDFS + SVM DGUFS + SVM UFSOL + SVM	Python	Radiomics	Hospital data of breast cancer patients	AUC= 0.72 SEN=70% SPE=74%	High performance in predicting cancer tumors with high indices
Park EK et al [52] Korea 2019	Predicting the status of breast cancer biomarkers	DT NB RF SVM ANN	Python	Radiogenomics	Hospital data of breast cancer patients	AUC=0.86 ACC=78%	Achieving accuracy and high efficiency in predicting the status of breast cancer biomarkers
Nguyen L et al [53] France 2018	Predicting response to drug therapy in breast cancer patients	RF-OMIC RF RF-all	R	Pharmacogenomis	Hospital data of breast cancer patients	SPE= 90%	Acceptable efficacy in predicting response to drug therapy in breast cancer patients
Dutta K et al [54] USA 2021	Prediction of response to treatment of breast cancer patients	U-Net, dense U-Net, Res-Net, recurrent residual UNet (R2UNet), and dense R2U-Net (D-R2UNet) CNN-based	MATLAB	Radiomics	Hospital data of breast cancer patients	AUC= 0.975 SEN=90% SPE=78%	High performance in predicting response to drug therapy in breast cancer patients
Zhang Y et al. [55] China 2021	Prognosis of pathological status of breast cancer in response to treatment	XGBoost, LASSO	Python	Radiogenomics	Data from the Breast Cancer Research Center	AUC= 0.87	Acceptable efficiency in predicting the pathological status of breast cancer in response to treatment
Chen J et al. [56] China 2022	Prediction of response to neoadjuvant treatment of breast cancer patients	Lasso, RR, ENR, SVM, RF, NNet1 NNet2, NNet3	Python	Genomics	IMMPORT, GSE163882 GSE123845	AUC=0.801 AUC=0.77	Acceptable efficacy in predicting response to drug therapy in breast cancer patients
Caballo M et al [57] Netherland 2020	Screening of tumor status of breast cancer patients	GAN U-net	MATLAB	Radiomics	National Cancer Institute patient data	SEN=92% SPE=93%	Acceptable efficacy in diagnosing tumor screening status
Pang T et al [58] Malaysia 2021	Tumor classification of breast cancer patients	GAN CNN	Python	Radiomics	Local and hospital data of breast cancer patients	SEN=88% SPE=86% ACC=90.4%	Acceptable efficacy in breast cancer classification
Ma S et al [59] USA 2016	Prognosis of biomarkers affecting the tumor of breast cancer patients	SVM RFE SVM, NB LR RF	R	Multi-omics	TCGA	AUC= 0.725	High efficiency in predicting the status of biomarkers on patients’ tumors
Braman NM et al. [60] USA 2017	Prediction of response to neoadjuvant treatment of breast cancer patients	LDA (DLDA) NB SVM	MATLAB	Radiomics	Local and hospital data of breast cancer patients	AUC= 0.83	High efficiency in predicting patients’ response to chemotherapy
Cui H et al [61] China 2023	Prediction of HER2+ receptor status in breast cancer patients	CNN, SVM, RF, DT, LR Naive Bayes, ANN K-NN	Python	Radiogenomics	Local and hospital data of breast cancer patients	AUC= 0.80	High performance prediction of HER2+ receptor status of breast cancer
Tyanova S et al. [62] Germany 2016	Classification of breast cancer tumors	SVM	R	Proteomics	Local and hospital data of breast cancer patients	AUC= 0.94	High performance in classifying breast cancer tumors
Yanovich G et al [63] Israel 2018	Classification of breast cancer tumors	K-means clustering	R	Proteomics	Local and hospital data of breast cancer patients	AUC= 0.75	High performance in classifying breast cancer tumors
Wang Z ET AL [64] China 2021	Prognosis of biomarkers affecting the tumor of breast cancer patients	BoostCI PCRM En-Cox LASSO-Cox MDNNMD DeepCorrSurv GPDBN	R	Genomics	TCGA	AUC=0.81 SEN=91% SPE=95% ACC= 90%	High efficiency in identifying the effectiveness of biomarkers in breast cancer tumors
Azzouz FB et al [65] France 2021	Prediction of triple negative breast cancer tumor type	GB RF XGB	Python	Transcriptomics	Genomics dataset	ACC=90%	High performance in predicting triple negative breast cancer tumor type

In 11 studies, the Cancer Genome Atlas (TCGA) was used as the data set source. Of these, four studies using CNN achieved high indices in predicting survival and recurrence of breast cancer. This indicates that the designed deep learning networks are superior in terms of comprehensive evaluation over traditional methods. Five studies using machine-learning models such as RF, SVR, and DNN in predicting the response to chemotherapy drugs in these patients reported the desirable performance of these drugs considering the type of tumor and its receptors. These models can be used to predict drug response for some specific drugs and potentially play a complementary role in personalized medicine. Two studies that analyzed and predicted cancer biomarkers on tumor growth in patients using SVM, RF, RE models reported the impact of each with high accuracy. The proposed algorithm improves the cost-effectiveness and accuracy of the screening process compared to current clinical guidelines. In two studies that used machine-learning models such as LASSON, ELASTIC NET, and RR on pharmacogenomics data from the Cancer Cell Line Encyclopedia (CCLE) to predict responsiveness to breast cancer treatment, the area under the ROC curve of the models indicated the desirable performance of the drug on patients. The proposed approach has the potential to enable the design of new hypotheses, improve drug selection, and lead to improvements in patient genomic-based treatments for cancer. Seven studies also analyzed the effect of chemotherapy drugs on drug-sensitive genomic data in cancer (GDSC) using machine learning and deep learning models, each of which reported high indices for their study. In other words, these models provide new methods for predicting anticancer drugs in human tissues and outperform human experts in predictive accuracy. Based on the effectiveness indices, in a large number of selected articles, methods based on SVM and RF, which are linear models, effectively predicted and diagnosed cancer with voluminous genomic data and a high number of feature parameters. Another algorithm used in radiomics articles was the convolutional neural network (CNN), a non-linear deep learning technique that can take an input image and is designed to improve automatic accuracy and provide acceptable efficiency in predicting the impact of pre-surgery chemotherapy (Table 3). In eight studies, the radiomics and multi-omics signature model provided better classification performance using linear and non-linear artificial intelligence methods, with SVM having a higher frequency, which has high accuracy in analyzing complex and voluminous data, compared to radiologists. The striking predictive ability of the radiomics signature is effective for responding to patient treatment.

**Table 3:** Distribution of applied AI algorithms and their categorizations by frequencies

Frequency		
**Linear and nonlinear models**		
RF		**15**
CART		1
DT		5
K-means		2
K-NN		4
MLP		1
LASSO		5
LR		2
LR Naive Bayesian		1
NB		**6**
SVM		**17**
SVM RFE		1
SVM-RRF		1
VNN		1
XGBoost		4
ANN		4
**Deep learning model**		
CNN		**9**
Dense U-Net		1
Dense2U-Net		1
DGUFS + SVM		1
DNN		1
Elastic Net		**4**
ENLIGHT CNN		1
GAN		2
MobileNetV3		1
NNet1		3
R2UNet		1
Res-Net		1
Res-MLP		1
ResNet-101		1
ResNet50		2
U-Net		2
UDFS + SVM		1
UFSOL + SVM		1
VGG16		1
VGG19		1

**Fig. 2:**
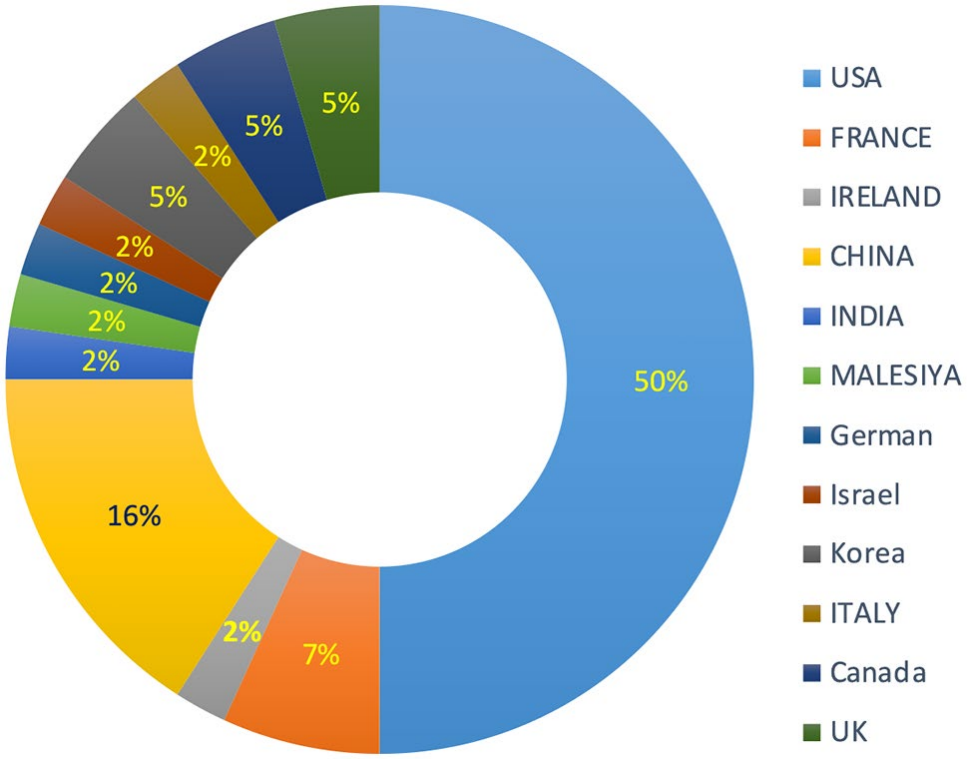
Frequency of paper in any country

**Fig. 3:**
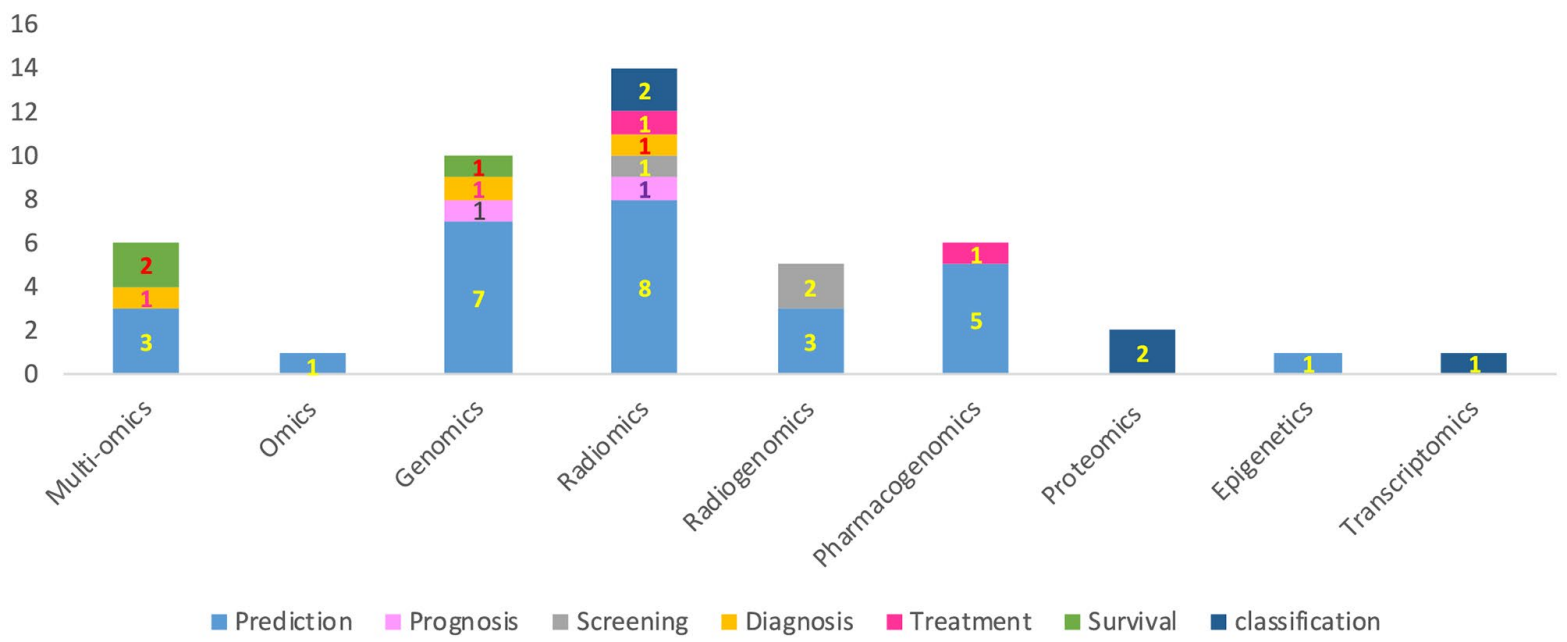
The distribution of citation by inputs and type of care

Also, the patterns obtained in radiomics can predict the occurrence of metastasis and response to treatment after neoadjuvant with high indices, the result of which is the selection of appropriate treatment for the patient. Twelve studies that used deep learning techniques on multi-omics, genomics, pharmacogenomics data to predict survival and diagnosis of breast cancer and responsiveness to treatment showed that the proposed policy has this potential with the appropriate selection of drugs, to provide the effectiveness of genomic treatments for breast cancer and has the ability to extract vital data and estimate predictive indices. This model can be used to predict drug response for some specific drugs and potentially play a complementary role in personalized medicine. It can also be a useful tool for determining the translation of gene expression signatures and predicting the status of breast cancer biomarkers on radiogenomics data in clinical decisions for personalized medicine. Most of the studies conducted were for the United States (The results showed that most articles were published in China and the United States and the number of articles published in the field of precision medicine has increased significantly in recent years) (Fig. 2) and the final classification of studies based on the type of activity performed for the personalized management of breast cancer is shown in (Fig. 3). The level of bias in 43 studies included in this review was diagnosed as low risk. Only two citations with medium bias risk [45, 46] and one with high bias risk [55] were evaluated. The questions “Were confounding factors identified?” and “Were there strategies to deal with confounding factors?” were not applicable in our entered studies, as our studies were not experimental.

## Discussion

Hopes for precise pharmacological treatment strategies in breast cancer (BC) and triple-negative breast cancer (TNBC) have been raised by the development of next-generation sequencing technologies, since breast cancer is a heterogeneous disease with various molecular types (e.g., HER2 + and TRPN, or estrogen or progesterone receptor). It is crucial to customize effective treatments for every patient due to the heightened risk of disease recurrence and mortality. Novel and efficacious treatments for metastatic breast cancer have been developed as a result of recent developments in precision medicine. Treatment for each patient is tailored using genomic testing to find genetic mutations that contribute to the growth of breast cancer [66]. Patients with positive BRCA1, 2 gene mutations can avoid metastasis by using targeted therapies that specifically target these genetic mutations. Immunotherapy is another instance of how precision medicine is used to treat metastatic breast cancer. Furthermore, the development of endocrine therapies hormones that promote the growth of breast cancer cells has been aided by precision medicine. A non-invasive procedure called liquid biopsy uses a patient’s blood sample to detect cancer genes or cells. This makes it possible to identify any new mutations that might arise during treatment and to monitor the progression of the disease in a minimally invasive manner. Another area of advancement [67, 68]. Medical decisions are usually associated with various and multiple variables, which make decision-making difficult. For example, oncologists have to combine a large volume of clinical, biological, genome and imaging data to achieve appropriate treatments, while their cognitive capacity can only integrate up to five factors (senses). Therefore, artificial intelligence can facilitate decisions that rely on multiple and diverse variables.

In the present study, 46 articles were selected with the aim of determining the application of artificial intelligence in personalized management of breast cancer. The goal was to select studies that focused on diagnosis, treatment, screening, prognosis, and prediction of disease in breast cancer patients. The aim of 22 studies was to predict the response to treatment and survival of patients. These studies, which had used various types of deep learning techniques, presented high AUC indices, which could indicate that the use of artificial intelligence in predicting the response to treatment and survival of patients has a high ability and this has increased the confidence of researchers. Machine learning techniques such as RF, SVM, XGBoost, which were used to investigate the response to chemotherapy on Pharmacogenomics data of patients, showed that with 100% sensitivity and an average AUC of 0.9, they could predict this process [21]. Therefore, this predictive ability can help doctors and scientists to use effective and alternative drugs for effective treatment of patients. Since predicting the response to neoadjuvant chemotherapy in breast cancer is of high importance and it has been seen that 15% of patients respond negatively to this type of treatment, studies have shown that deep learning techniques such as CNN and VGG16 for predicting the response to neoadjuvant treatment on pathological images and omics data of patients had high index results (SEN = 98%, AUC = 1) [23, 40]. Therefore, with the automation of analysis and reviews, the speed of image analysis increases and the error rate of doctors and specialists decreases. Also, in a study to screen patients, one of the CNN models named U-Net was used to analyze the radiomics data of patients, which had presented 92% and 93% sensitivity and accuracy, respectively. The findings of the measured indices showed that machine learning can also be effective in screening patients. Considering the positive effects of artificial intelligence, one of the challenges of using artificial intelligence in personalized medicine is the lack of available and high-quality data and the lack of participation of the most important variables in modeling, which can lead to the identification of unrelated patterns. On the other hand, ensuring privacy and data security, maintaining ethical considerations are other challenges of using artificial intelligence in analyzing patient data, which artificial intelligence technology in block-chain can increase data management, privacy by facilitating the storage and secure sharing of patient records, medical research data and other sensitive information [69].

To this end, we argue that one of the challenges that medicine faces in personalized management of breast cancer is the problem of drug resistance in patients, which requires looking for alternative treatments, which fortunately artificial intelligence can help doctors in this field [70, 71]. The use of artificial intelligence and analytical techniques can provide new models for predicting the response to disease treatment and be effective in helping doctors choose appropriate personalized treatments by using them in medical decision support systems [72, 73]. Although this research was able to illustrate the artificial intelligence techniques used in breast cancer management, we faced some limitations in conducting this research, one of which was the lack of inclusion of some articles and studies presented at conferences that we did not have access to their full texts. We also only used English articles, so there is a possibility of losing several relevant studies and articles with effective results in non-English languages.

## Conclusion

Findings of the present study show that the use of machine learning in the fields of prognosis, diagnosis, prediction, treatment, and screening, which collectively emphasize breast cancer management, have had an effective role, and it can be hoped that the growth of artificial intelligence in the not-too-distant future will provide a very high confidence to healthcare providers to solve patients’ problems. The focus and emphasis on the use of deep learning is not only the recommendation of researchers in the field of breast cancer management with the help of artificial intelligence, but also the present study emphasizes this recommendation. Simultaneously with the integration of patient-specific data and medical knowledge, artificial intelligence systems can provide optimal treatment options and predict treatment outcomes. This capability can help health care providers in making more informed decisions and improving patient care. It can also lead to faster diagnosis, reduced waiting time, faster patient recovery, and ultimately increased efficiency of health care. Following more effective treatment, reduced side effects and improved patient satisfaction, the possibility of discovering new biomarkers and treatment methods are other effects of it. New policies, preventive tactics, diagnosis, and treatment for the appropriate person at the appropriate time will need to be guided by innovative research combined with data science, as well as innovative diagnostic systems for equitable and safe data sharing. One factor to take into account is the accessibility of knowledge in remote areas, particularly the availability of qualified experts when needed. Many examples of enhanced diagnostic capabilities in resource-poor settings, which could result in better patient classification and, ultimately, more individualized treatment planning, have been made possible by artificial intelligence. This feature has the potential to improve patient care by assisting healthcare professionals in making better decisions. Additionally, it may result in quicker patient recovery, a shorter waiting period, quicker diagnoses, and ultimately more efficient health care delivery. There is no doubt that investing in AI now will pay off later on in the form of improved population health and cost savings from precision medicine. In precision public health and medicine, governments are essential because they facilitate the equitable application of knowledge to the development of evidence-based policies, procedures, and environmental modifications. Through error reduction and the potential to significantly reduce the number of missed cancer diagnoses, artificial intelligence offers rich opportunities for designing intelligent systems and medical decision support, thereby creating new services.

## Data Availability

The datasets used and/or analyses during the current study available from the corresponding author on reasonable request. Declarations Ethics approval and consent to participate Not applicable.

## Abbreviations

ER: Estrogen receptors
DNA: deoxyribonucleic acid
SPICE: setting, perspective, intervention, comparison, and evaluation
PICO: Population, Intervention, Comparison and Outcomes
ACC: accuracy
SEN: sensitivity
SPE: specificity
ROC: Receiver operating characteristic
AUC: Area under the ROC curve
MRI: Magnetic resonance imaging
PET/CT: positron emission tomography
HER2: Human epidermal growth factor receptor-2
PR: progesterone receptor
TCGA-LGG: The Cancer Genome Atlas Low Grade Glioma
VGG: Visual Geometry Group
RR: Ridge regression
LASSO: least absolute selection and shrinkage operator
DT: Decision tree
NB: Naive Bayesian
RF: Random Forest
LR: Logistic Regression
DNN: Deep neural network
UDF: User defined functions
SVM: Support Vector Machine
HMF: hydroxyl methyl furfural
VNN: Volterra Neural Network
ANN: Artificial Neural Network
SVR: Support Vector Regression
TCGA: the Cancer Genome Atlas
CNN: Convolutional neural network
NLP: Natural Language Processing
KNN: K-nearest neighbor algorithm
CCLE: Cancer Cell Line Encyclopedia
DeepPT: Deep Pathology for Treatment
RBM: Restricted Boltzmann machine
DGUFS: Unsupervised Feature Selection
GAN: Generative adversarial network
ROC: Receiver Operating Characteristic
XGBoost: extreme Gradient Boosting Tree
GDSC: Genomics of Drug Sensitivity in Cancer
UFSOL: Unsupervised Feature Selection with Ordinal Locality
